# Multi-method simulation modelling of circular manufacturing systems for enhanced decision-making

**DOI:** 10.1016/j.mex.2022.101709

**Published:** 2022-04-22

**Authors:** Malvina Roci, Niloufar Salehi, Saman Amir, Farazee.M.A. Asif, Sayyed Shoaib-ul-Hasan, Amir Rashid

**Affiliations:** a Department of Production Engineering, KTH Royal Institute of Technology, Stockholm, Sweden; b Department of Marketing & Strategy and Centre for Sustainability Research, SSE Stockholm School of Economics, Stockholm, Sweden

**Keywords:** Circular economy, Circular manufacturing systems, Multi-method simulation, Complex adaptive systems, Agent-based, Discrete-event, System dynamics, CMS:, Circular Manufacturing Systems, AB:, Agent-based, DE:, Discrete-event, SD:, System dynamics, GIS:, Geografic Information System, GHG:, Greenhouse gas emissions

## Abstract

Circular manufacturing systems (CMS) constitute complex value networks comprising a large and diverse set of stakeholders that collaborate to close the loop of products through multiple lifecycles. Complex systems modelling and simulation play a crucial role in providing quantitative and qualitative insights into the behaviour of such systems. In particular, multi-method simulation modelling that combines agent-based, discrete-event, and system dynamics simulation methods is considered more suitable to model and simulate CMS as it allows to capture their complex and dynamic nature. This paper provides a step-by-step approach on how to build a CMS multi-method simulation model in order to assess their economic, environmental, and technical performance for enhanced decision-making. To model and simulate CMS three main elements need to be considered:

• A multi-method model architecture where the CMS stakeholders with heterogeneous characteristics are modelled individually as autonomous agents using agent-based, discrete-event, and system dynamics.
• An agent environment defined by a Geographic Information System (GIS) to establish connections based on agents’ geographic location.
• The product journey resulting from the product's interaction with various CMS stakeholders in the circular value network is traced throughout its multiple lifecycles.

## Graphical abstract

**Figure:**
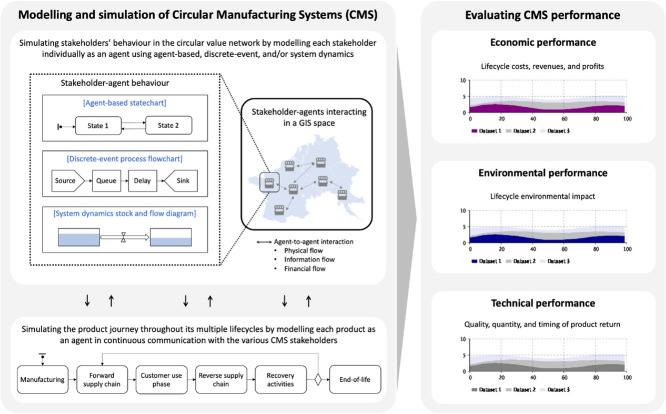

**SPECIFICATIONS TABLE**

**Table:** 

Subject Area;	Environmental Science
More specific subject area;	*Circular economy, complexity science*
Method name;	*Multi-method simulation modelling of circular manufacturing systems*
Name and reference of original method;	*Agent-based; Discrete-event; System dynamics*
Resource availability;	*A simulation development platform that combines simulation features of agent-based, discrete-event, and system dynamics modelling approaches (e.g., AnyLogic) is recommended for reproducing the method.*

## Background

Circular manufacturing systems (CMS) refer to systems that are designed intentionally to close the loop of materials and products through multiple lifecycles [7]. These systems can be viewed as complex adaptive systems composed of an interconnected network of a large and diverse set of stakeholders that collaborate to close the loop of products through multiple lifecycles [8]. Complex systems modelling and simulation is becoming an increasingly popular approach to analyse the behaviour of complex systems as this approach allows to capture non-linear behaviour as well as time and casual dependencies [6]. The major simulation modelling paradigms employed to simulate complex systems are system dynamics (SD), discrete-event (DE), and agent-based (AB) [2]. SD is mostly employed for strategic modelling as this modelling approach operates at a high abstraction level; DE takes a process-centric view of the system and supports medium and medium-low abstraction; whereas AB takes a decentralized and individual-centric approach to the system by describing the system as interacting objects with their own behaviours [2]. Moreover, SD deals with continuous processes, whereas DE and AB work mostly in discrete time, i.e., the system has state changes in discrete points of time.

Multi-method simulation modelling, i.e., a combination of the aforementioned simulation modelling methods, provides a higher level of flexibility for modelling and simulating complex systems as it allows to take into account the complementary capabilities and recognize the limitations of each modelling method [4,12]. Thus, multi-method simulation modelling is considered more suitable to model and simulate CMS as it allows to capture their complex and dynamic nature [1,8]. As different multi-method model architectures can be built by combining the different modelling approaches, the authors presented in Roci et al. [8] a multi-method model architecture to model and simulate CMS. By considering CMS as a complex value network composed of many stakeholders engaged in achieving circularity as a collective outcome, the authors proposed a multi-method model architecture where the different CMS stakeholders are modelled individually as autonomous agents using AB, DE, and SD modelling methods. Thus, this approach allows for characterising and analysing CMS by modelling the system as a collection of autonomous decision-making entities with their own behaviours and relationships. This paper provides a step-by-step approach on how to build a CMS multi-method simulation model using the multi-method model architecture proposed by the authors in Roci et al. [8]. In addition, as a simulation development platform that supports AB, DE, and SD simulation methods is needed to implement and reproduce the CMS multi-method simulation model, this paper provides the detailed procedure to construct the computer program using the case study of a white goods manufacturer.

## Method details

**Fig. 1:**
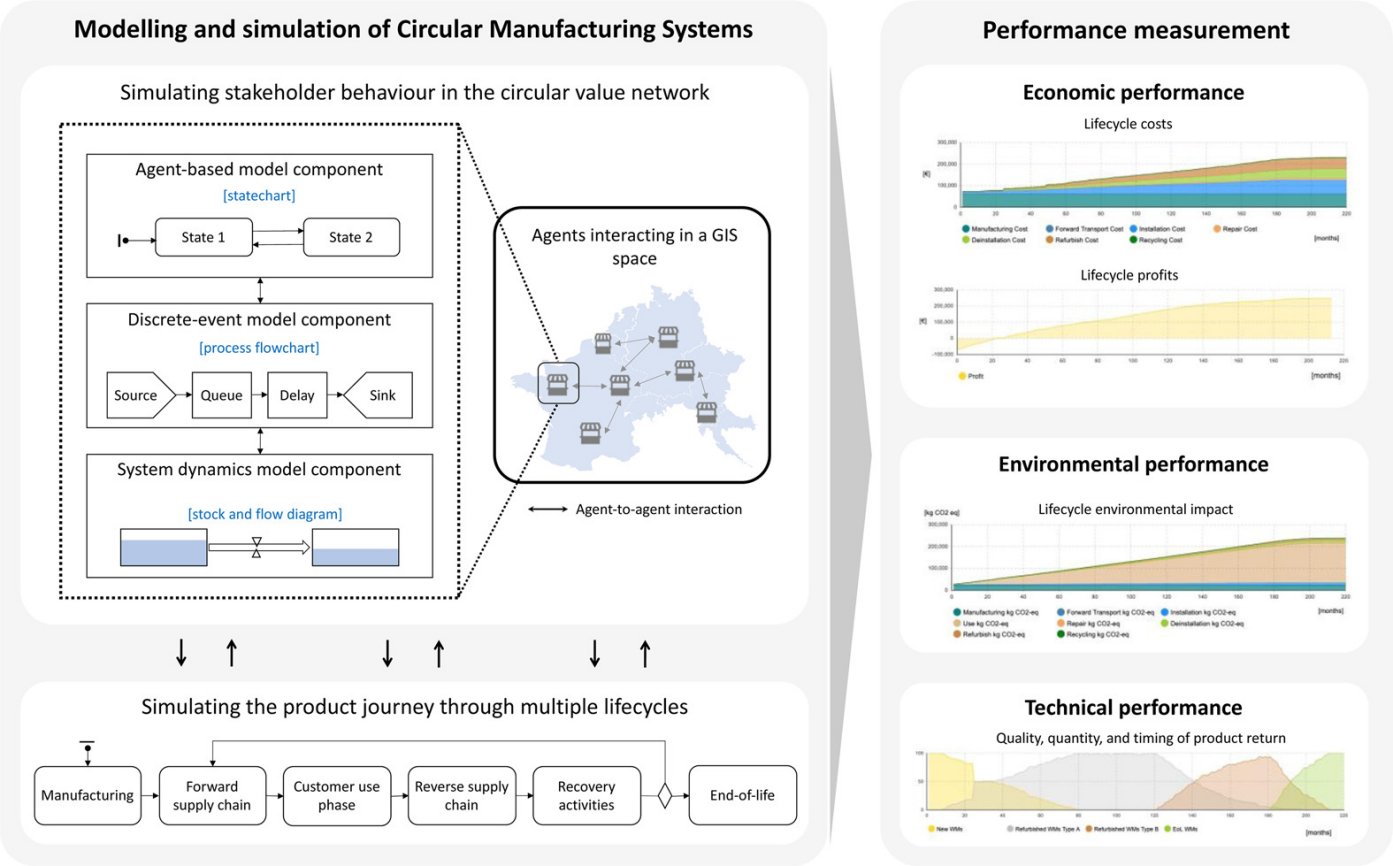
Multi-method simulation modelling of circular manufacturing systems. The term agent represents a stakeholder in the circular value network placed in a Geographic Information System (GIS) space. Compiled from Roci et al. [8].

An overview of the CMS multi-method simulation model is shown in Fig. 1 and each step required to build this model is explained in detail below. Notice that the CMS multi-method simulation model is not a simple sequential process. As one proceeds with building the CMS simulation model, it is often desirable to combine the steps or go back to a previous step. For instance, while defining the behaviour of a CMS stakeholder-agent (i.e., step 1) it is preferable to set up the agent's interface (i.e., step 2) needed to establish the communication with other stakeholder-agents.

*Step 1 - Define the set of CMS stakeholder-agents and their behaviours*

The first step in building the CMS multi-method simulation model consists in defining the set of CMS stakeholder-agents and their behaviours. The set of CMS stakeholder-agents consists of all relevant value chain stakeholders involved in achieving circularity as an overall outcome (e.g., suppliers, manufacturers, service providers, customers, and value recovery entities). Given the heterogeneous nature of the CMS stakeholders, an AB statechart, a DE process flowchart, an SD stock and flow diagram, or a combination of these modelling constructs are employed to simulate the behaviour of each CMS stakeholder as shown in Fig. 1. Statecharts are an advanced construct in AB modelling used to simulate event-driven and time-driven agent behaviour [2]. Thus, for stakeholder-agents where event- and time-ordering of operations is very pertinent (e.g., customers), one can best characterize their behaviour by employing AB statecharts. Process flowcharts are an advanced construct in DE modelling used to adopt a process-centric view of the system. Thus, for stakeholder-agents where manufacturing, logistics, and reprocessing activities are relevant (e.g., manufacturers and service providers), one can best characterize their behaviour by employing DE process flowcharts [3]. Whereas, stock and flow diagrams represent an advanced construct in SD modelling used to simulate continuous processes [10] where stocks represent accumulations (e.g., stock of material, products, money) and flows define how these stocks change over time. Thus, this modelling approach can be employed to capture continuous system behaviour.

*Step 2- Define the set of relationships among CMS stakeholder-agents*

The second step in building the CMS multi-method simulation model consists in defining the relationships and interactions among the CMS stakeholder-agents. Unidirectional or bidirectional connections are employed to define how and with whom stakeholder-agents interact. These interactions among CMS stakeholders comprise exchange processes in terms of physical (e.g., products), information (e.g., order requests), and financial flows (e.g., revenue streams and cost streams) through time along the circular value chain. These connections between agents can be defined in simulation modelling by building the agent's interface [2], i.e., the set of attributes, methods, ports, or messages that other stakeholder-agents can employ to communicate and interact.

*Step 3 – Define the environment the CMS stakeholder-agents live and interact*

The third step in building the CMS multi-method simulation model consists in defining the simulation environment in which the stakeholder-agents will be placed. Among the different simulation *environments* available (e.g., discrete space, continuous space, or Geographic Information System (GIS) space), GIS space is more suitable for placing the CMS stakeholder-agents as it allows to consider real-life locations of the CMS stakeholders, thus, retrieve real distances travelled between CMS stakeholders to better estimate transport cost and greenhouse gas emissions. Thus, the different CMS stakeholder-agents are placed on a GIS map depending on their geographical location.

*Step 4 – Simulating the* product journey through its multiple lifecycles

As effective implementation of CMS requires full control of the product throughout all the stages of the value chain, the fourth step in building the CMS multi-method simulation model consists in simulating the product journey through its multiple lifecycles resulting from the product's interaction with the various CMS stakeholder-agent in the circular value network. For this purpose, each product is modelled as an individual agent in continuous communication with the CMS stakeholders throughout its lifetime.

*Step 5 – Measure system performance*

Finally, the last step in building the CMS multi-method simulation model consists in defining the set of variables needed for collecting statistics both at the agent level and at the system level. These statistics are used to assess the CMS performance in terms of economic (e.g., lifecycle costs, lifecycle revenues, and lifecycle profits), environmental (e.g., lifecycle environmental impact), and technical (e.g., quality, quantity and timing of product return) performance.

## Method implementation

A simulation development platform that supports AB, DE, and SD simulation methods is needed to implement the CMS multi-method simulation model. In this paper, the multi-method simulation model developed by the authors in Roci et al. [8] to model and simulate the CMS adopted by a white goods manufacturer implementing CMS in practice is used to illustrate the technical steps employed to construct the simulation model. The AnyLogic modelling software, a Java-based simulation development platform that supports AB, DE, and SD modelling methods, is used to implement the multi-method simulation model developed for the white goods manufacturer.

The white goods manufacturer is deploying white-goods-as-a-service for washing machines within the EU funded ReCiPSS project [5]. The washing machines are offered through a subscription-based scheme where customers can choose between Gold, Silver, and Bronze service packages depending on their preferences (e.g., service level, washing machine type, and subscription flexibility). The CMS stakeholders involved in this process of closing the loop of the washing machines through multiple lifecycles include the manufacturer, the service providers, the customers, and the value recovery entities. The manufacturer is responsible for manufacturing long-lasting washing machines. After manufacturing, the washing machines are delivered via forward transport to the service providers responsible for deploying the subscription-based offerings in the market. During the deployment phase, the service providers are responsible for repair and maintenance activities in case of washing machine failure, and perform the recovery operations (e.g., refurbishing) after each end-of-use. The service providers have a pool of technicians to perform the operational activities, e.g., installation, repair and maintenance, and deinstallation of the washing machines. When the washing machines reach their end-of-life, they are delivered to the local recycler for end-of-life treatment.

To build the multi-method simulation model of the white goods manufacturer, the first step consists in creating the agents responsible for the system behaviour. Three different agent categories are considered in this model: stakeholder-agents, product-agents, and order-agents. The stakeholder-agents represent the set of stakeholders responsible for closing the loop of washing machines through multiple lifecycles. These stakeholder-agents include the Manufacturer agent, the ServiceProvider agent, the Customer agent, and the Recycler agent. The product-agents represent the multiple lifecycles washing machines deployed through a subscription-based scheme denoted as Product agents, thus simulating the physical flow. Whereas the order-agents represent the agents responsible for establishing the communication between stakeholder-agents, thus simulating the information flow. These order-agents include the ProductRequest agent (i.e., ServiceProvider-to-Manufacturer agent communication), the ServiceRequest agent (i.e., Customer-to-ServiceProvider agent communication), and TreatmentRequest agent (i.e., ServiceProvider-to-Recycler agent communication).

In AnyLogic, one can create a single agent, a population of agents (i.e., a given number of agents of the same type), or an agent type only. In this case, the stakeholder-agents and the product-agents are created as a population of agents. While the stakeholder-agents populations contain a given number of agents, the Product agent population is initially empty and it is populated dynamically by the Manufacturer agent at model runtime when a new washing machine is manufactured. On the other hand, the order-agents (i.e., the ProductRequest agent, the ServiceRequest agent, and the TreatmentRequest agent) are created as agent types only because there are no agents of this type initially. Order-agents are created dynamically at model runtime when a stakeholder-agent places an order on another stakeholder-agent.

**Fig. 2:**
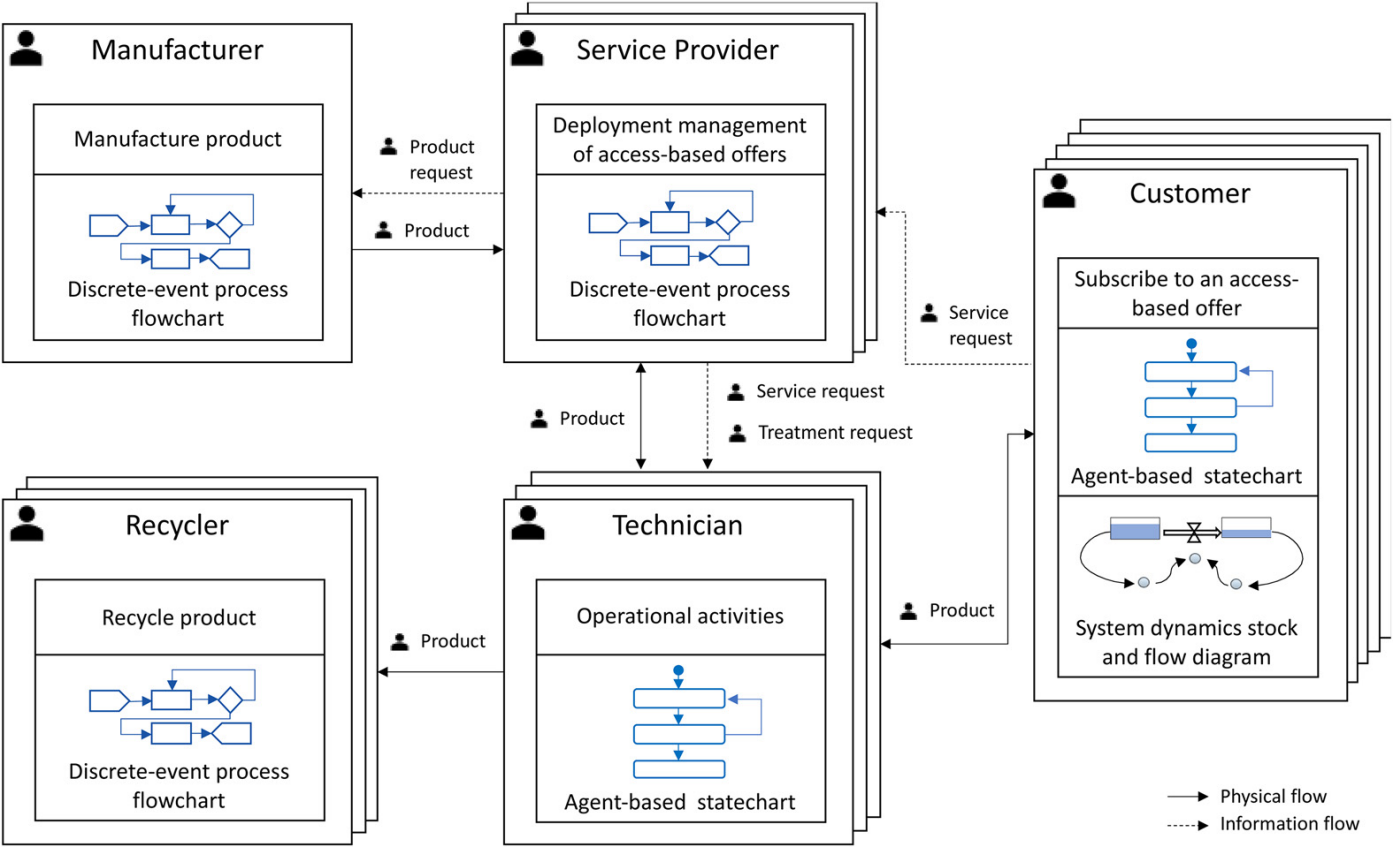
Overview of the CMS multi-method simulation model of the white goods manufacturer.

An overview of the agents developed to simulate the CMS adopted by the white goods manufacturer and their interrelations are shown in Fig. 2. DE process flowcharts are used to model the behaviour of the Manufacturer agent, the ServiceProvider agent, and the Recycler agent as these stakeholders deal with manufacturing, logistics, and reprocessing activities. Whereas AB statecharts are used to model the behaviour of the Customer agent, and the Technician agent, as for these stakeholders the event- and time-ordering of operations is very pertinent. In addition, SD stock and flows diagrams used to simulate continuous processes, are employed within the Customer agent to simulate the continuous revenue flows and greenhouse gas (GHG) emissions generated by each customer during the subscription period. The technical steps employed to simulate each CMS stakeholder and their interactions are explained in detail in the following sub-sections.

### Customer agent

**Fig. 3:**
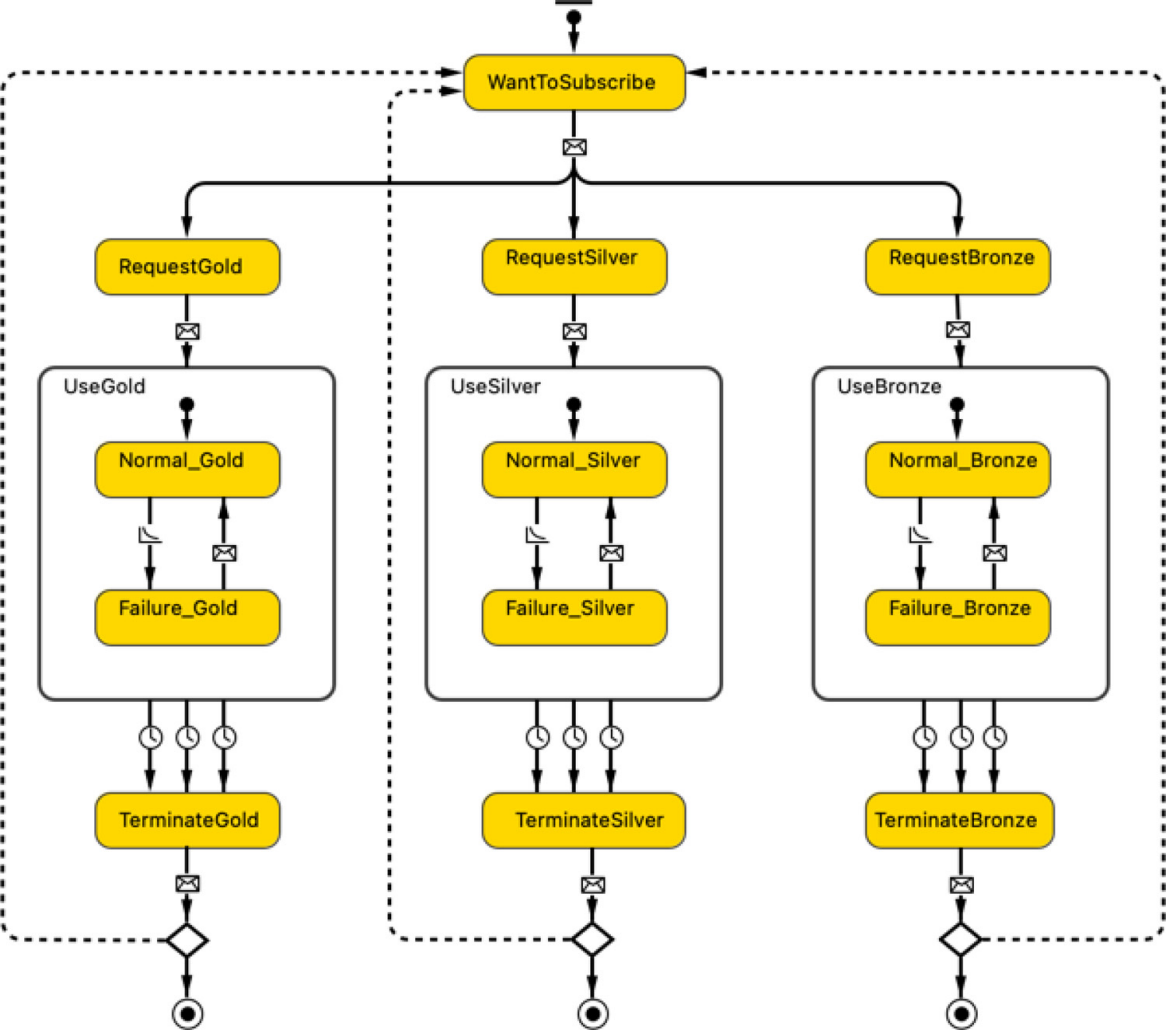
Agent-based statechart to simulate the behaviour of the Customer agent.

To simulate the behaviour of the Customer agent in a subscription-based scheme, first a set of attributes relevant to characterize the Customer agent is defined. In Java, an attribute is a variable that has its own type (e.g., int, double, String, Boolean) that is used to represent agent characteristics. A detailed list of the attributes employed to simulate the Customer agent is reported in Table 1. These attributes include a unique identifier number (ID), location, contract duration, monthly subscription fee, and the number of washing cycles per month. Second, the AB statechart shown in Fig. 3 is developed to define the behaviour of a customer in a subscription-based model as a sequence of states. When a new Customer agent is created, it enters the WantToSubscribe state. Depending on the service package preferred, the Customer agent enters the RequestGold, RequestSilver, or RequestBronze state. At this stage, an order-agent of type ServiceRequest is created to model the requests the Customer agent sends to the ServiceProvider agent. This ServiceRequest agent contains two main parameters: the reference to the customer who sends the request, and the request type (e.g., Gold, Silver, or Bronze). These service requests are created dynamically by customers at model runtime by calling the method requestSubscription(RequestTypetype)*.* A method is a block of code in java to perform a specific task or operation. The detailed definition of this method is reported in Table 1. Notice that RequestType is an attribute of type option list. Option lists are used to define agent attributes with a limited choice of alternative options. The available options for the Customer agent are Gold, Silver, and Bronze.

**Table 1:** Main attributes, methods, and agent links of the Customer agent.

Type	Name	Description
Attributes	ID: int	Unique customer identifier number of type integer
	country: String	Country of residence of type text strings
	region: String	Region of residence of type text strings
	serviceProvider: ServiceProvider	Service provider allocated to the customer of type ServiceProvider
	contractDuration: double	Contract duration of the subscription package of type double (i.e., a real number)
	subscriptionFee_Gold: double	Monthly subscription fee applied to a Gold customer.
	subscriptionFee_Silver: double	Monthly subscription fee applied to a Silver customer.
	subscriptionFee_Bronze: double	Monthly subscription fee applied to a Bronze customer.
	washingCycles_Gold: int	Number of washing cycles per month assigned to a Gold customer
	washingCycles_Silver: int	Number of washing cycles per month assigned to a Silver customer
	washingCycles_Bronze: int	Number of washing cycles per month assigned to a Bronze customer
	RequestType: option list	Option list to define the options available for a Customer agent, i.e., Gold, Silver, Bronze, Repair, and Terminate.
Methods	requestSubscription (RequestType type): void	Customized method developed to establish a communication with the ServiceProvider agent regarding subscription requests. This method is called when the Customer agent enters the WantToSubscribe state of the AB statechart shown in Fig. 3 to communicate with the ServiceProvider agent indicating the service package selected (i.e., Gold, Silver, or Bronze). The method is defined as follows: void requestSubscription(RequestType type) { ServiceRequest request = new ServiceRequest(this, type); send(request, ServiceProvider); } The Java keyword new creates a new instance of the agent type ServiceRequest by indicating first the reference to the Customer agent that sends the request, and then the request type (i.e., Gold, Silver, or Bronze). The Java keyword this is used to return the reference to the Customer agent that sends the request. Whereas, the Java keyword void denotes that the method does not have a return type.
	requestService (RequestType type): void	Customized method developed to establish a communication with the ServiceProvider agent regarding service requests. This method is called on the following occasions: - The Customer agent is in the use phase state of the AB statechart shown in Fig. 3 (i.e., UseGold, UseSilver, UseBroze) and communicates with the ServiceProvider agent to request a repair service in case of product failure (i.e., Failure_Gold, Failure_Silver, or Failure_Bronze). - The Customer agent is in the terminate subscription state of the AB statechart shown in Fig. 3 (i.e., TerminateGold, TerminateSilver, TerminateBroze) and communicates with the ServiceProvider agent to request a subscription termination. The method is defined as follows:void requestService(RequestType type) {ServiceRequest request = new ServiceRequest(this, type);send(request, ServiceProvider);}The Java keyword new creates a new instance of the agent type ServiceRequest by indicating first the reference to the Customer agent that sends the request, and then the request type (i.e., Repair, or Termination). The Java keyword this is used to return the reference to the Customer agent that sends the request. Whereas, the Java keyword void denotes that the method does not have a return type.
	time(): double	Built-in AnyLogic method that returns the current model time in model time units. This method is used to account for the time a Customer agent enters and exits the use phase state (i.e., UseGold, UseSilver, and UseBroze of the AB statechart shown in Fig. 3), thus, estimating the subscription duration. Notice that the model time unit is set to be months.
	inState(StatechartState state): boolean	Built-in AnyLogic method that returns true if the agent is in the specified state of its statechart. This method is called in the system dynamics flow elements (i.e., flowGold, flowSilver, and flowBronze of the SD stock and flow diagram shown in Fig. 4) to test if the Customer agent is currently in the use phase state (i.e., UseGold, UseSilver, and UseBronze of the AB statechart shown in Fig. 3) to account for its monthly revenue flows and kg CO2-eq emissions. Notice that this method call connects the agent-based module to the system dynamics module within the Customer agent.
Variables	wm: Product	Variable used during model runtime to trace and track the washing machine received by the Customer agent.
	durationGold: double	Variable used during model runtime to store the subscription duration of a Gold customer. This is achieved by calling the method time()when the customer enters and exits the UseGold state.
	durationSilver: double	Variable used during model runtime to store the subscription duration of a Silver customer. This is achieved by calling the method time() when the customer enters and exits the UseSilver state.
	durationBronze: double	Variable used during model runtime to store the subscription duration of a Bronze customer. This is achieved by calling the method time() when the customer enters and exits the UseBronze state.
	promptService: boolean	Variable used during model runtime to store the service readiness preferred by the customer. It returns true if prompt service is preferred, and false otherwise.
	revenuesGold: double	Variable used during model runtime to store the revenues generated by a Gold customer.
	revenuesSilver: double	Variable used during model runtime to store the revenues generated by a Silver customer.
	revenuesBronze: double	Variable used during model runtime to store the revenues generated by a Bronze customer.
	emissionsGold: double	Variable used during model runtime to store the kg CO2-eq generated by a Gold customer as a result of the total number of washing cycles.
	emissionsSilver: double	Variable used during model runtime to store the kg CO2-eq generated by a Silver customer as a result of the total number of washing cycles.
	emissionsBronze: double	Variable used during model runtime to store the kg CO2-eq generated by a Bronze customer as a result of the total number of washing cycles.
Agent links & communications	Bi-directional links with the ServiceProvider agent, *Customer* and the Product agent. These links allow agent communications via message passing and inter-agent method calls.

Upon reception of a subscription request, the ServiceProvider agent sends a technician to install the washing machine at the customer's location. The Custome*r* agent, thus, enters the use phase state (i.e., UseGold, UseSilver, or UseBronze). During this stage, the Customeragent is in communication with the ServiceProvider agent in case the washing machine breaks down (i.e., Failure_Gold, Failure_Silver, or Failure_Bronze) by calling the method requestService(RequestTypetype) with RequestType being Repair. When the Customer agent intends to terminate the subscription, it enters the terminate subscription state (i.e., TerminateGold, TerminateSilver, or TerminateBronze) where the method requestService(RequestTypetype) is called again, with the request type being Termination. Notice that when the method requestService(RequestTypetype) is called in the AB statechart, an order-agent of type ServiceRequest is created to model the requests the Customer agent sends to the ServiceProvider agent by indicating the reference to the customer who sends the request, and the request type (e.g., Repair or Termination).

**Fig. 4:**
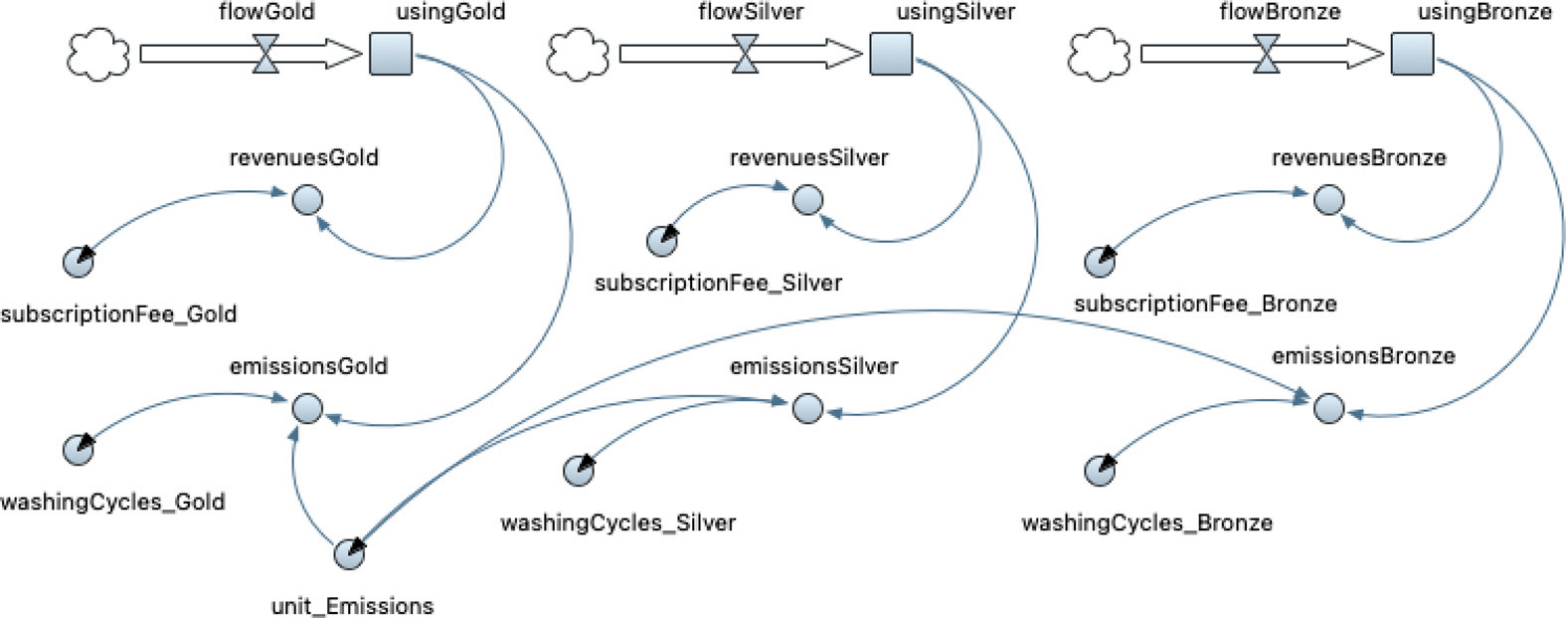
System dynamics stock and flow diagram to simulate revenue flows and emissions generated by the Customer agent during the use phase stage.

In an AB statechart, the model time stays constant during an event execution and is only advanced between events [2]. Thus, the exact duration of the use phase state can be estimated only when the Customer agent exits the state. However, given the continuous nature of subscription-based business models, it is important to access the use phase state on a continuous-time unit basis (i.e., from the moment the Customer agent enters the subscription period until it terminates it). In this way, it is possible to assign user behaviour data (e.g., washing cycles) and extract statistics (e.g., revenues and GHG emissions) on a time unit basis instead of aggregated values based on the total subscription period. To capture this time continuity, an SD modelling approach is employed due to its continuous nature. Thus, the SD stock and flow diagrams shown in Fig. 4 is developed to simulate the continuous revenue flows and GHG emissions generated by a Customer agent during the subscription period. The stock usingGold, usingSilver, and usingBronze are used to represent the subscription duration in time units (i.e., months) of a Gold, Silver, and Bronze customer respectively. These stocks are linked to the AB statechart shown in Fig. 3 by calling the conditional operators inState(UseGold)?1:0 in the SD flowGold element, inState(UseSilver)?1:0 in the SD flowSilver element, and inState(UseBronze)?1:0 in the SD flowBronze element. This conditional operator inState(StatechartStatestate)?1:0 returns one if the Customer agent is in the specified statechart state and zero otherwise. Thus, while the customer is in the use phase state, the value of the stock is incremented by one every time unit. As a result, the revenues generated from each customer-agent every month are estimated given the monthly subscription fee defined by the attributes subscriptionFee_Gold, subscriptionFee_Silver, and subscriptionFee_Bronze. In addition, the GHG emissions during the use phase state are estimated by taking into account the number of washing cycles a customer uses during its subscription period defined by the attributes washingCycles_Gold, washingCycles_Silver, and washingCycles_Bronze for a Gold, Silver, and Bronze customer respectively and the GHG emissions per washing cycle defined by the attribute unit_Emissions.

The main attributes, methods, and agent links used to develop the model logic of the Customer agent are reported in Table 1.

### Service provider agent

**Fig. 5:**
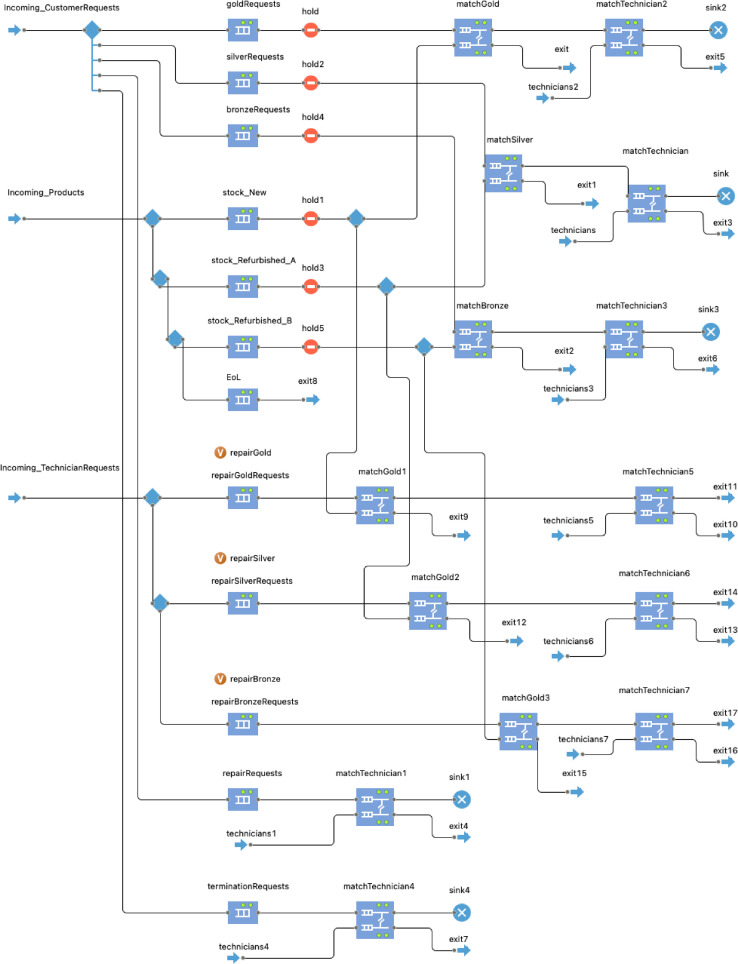
Discrete-event process flowchart to simulate service management at the service provider.

A discrete-event modelling method is employed to simulate the behaviour of the ServiceProvider agent as this stakeholder deals mainly with service management activities. The DE process flowchart shown in Fig. 5 is developed to simulate the deployment management of subscription-based offerings from the ServiceProvider agent by using a sequence of operations performed over entities. The two types of entities handled by the ServiceProvider agent are the customer requests and products. As mentioned earlier, both customer requests and products are modelled as agents, (i.e., the ServiceRequest agent and the Product agent). In multi-method simulation modelling, agents become entities that enter the DE process flowchart to be processed [2]. Thus, when a request is received by a customer, it enters the Incoming_CustomerRequests block of the DE process flowchart shown in Fig. 5. Depending on the request type, the incoming customer requests are routed to different queues awaiting to be handled in the DE process flowchart.

The ServiceProvider agent is in communication with the Manufacturer agent for ordering new washing machines. This process is modelled by creating a new order-agent of type ProductRequest that contains two main parameters: the reference to the service provider that sends the request, and the treatment type (e.g., Manufacture). These product requests are created dynamically by the service provider at model runtime by calling the method requestManufacturer(TreatmentTypetype)*.* Note that TreatmentType is an attribute of type option list defined for agent attributes with a limited choice of alternative options. The available options for the ServiceProvider agent are Manufacture, Repair, Refurbish, and Recycle. In this model, it is assumed that the service provider performs the repair and refurbish activities itself. However, the model has the capability of requesting these treatment types from another agent. Regarding the recycling of the products, the ServiceProvider agent is in communication with the Recycler agent. This process is modelled by creating a new order-agent of type TreatmentRequest that contains two main parameters: the reference to the service provider that sends the request, and the treatment type (e.g., Recycle). These treatment requests are created dynamically by the ServiceProvider agent at model runtime by calling the method requestRecycler(TreatmentTypetype)*.*

In addition, the ServiceProvider agent has a pool of technicians that are reasonable for the installation, repair, and deinstallation of the washing machines. These technicians are modelled as agents with their own behaviour as shown in the next sub-section.

The main attributes, methods, and agent links used to develop the model logic of the ServiceProvider agent are reported in Table 2.

**Table 2:** Main attributes, methods, and agent links of the ServiceProvider agent.

Type	Name	Description
Attributes	country: String	Geographical location of the service provider indicating the country
	region: String	Geographical location of the service provider indicating the region
	initialStockNew: int	Initial stock of new washing machines of type integer
	initialStockRefurbished: int	Initial stock of refurbished washing machines of type integer
	nrTechnicinas: int	Number of technicians for service deployment and management of type integer
	serviceTransportCost: double	Transport cost expressed in €/km/van used to transport the washing machines to/from customers’ location. It is assumed that a light commercial vehicle is used for the transportation of the washing machines.
	serviceTransportCO2: double	Transport kg CO2-eq expressed in kg CO2/tkm due to the transport of the washing machines to/from customers’ location. It is assumed that a light commercial vehicle is used for the transportation of the washing machines.
	labourCost: double	Labour cost regarding installation, repair, and deinstallation activities performed by the technicians
	repairCost: double	Repair cost of the washing machine including labour cost and spare parts replacement
	repairCO2: double	Kg CO2-eq due to repair of the washing machine
	refurbishCost: double	Refurbish cost of the washing machine
	refurbishCO2: double	Kg CO2-eq due to refurbish of the washing machine
	TreatmentType: option list	Option list to define the options available for the ServiceProvider agent, i.e., Manufacture, Repair, Refurbish, Recycle.
Methods	request Manufacturer (Treatment Type type): void	Customized method developed to establish a communication with the Manufacturer agent to request the manufacturing of new washing machines. It is called when the initial stock of new washing machines reaches a determined threshold (i.e., the safety stock). The method is defined as follows: void request Manufacturer (Treatment Type type) { Product Request productRequest = new Product Request (this, type); send (product Request, Manufacturer); } The Java keyword new creates a new instance of the agent type ProductRequest by indicating first the reference to the ServiceProvider agent that sends the request, and then the request type (i.e., Manufacture). The Java keyword this is used to return the reference to the ServiceProvider agent that sends the request.
	findTechnician(): Technician	Customized method developed to find a Technician agent available to deploy a service (e.g., installation, repair, or deinstallation). The method is defined as follows: Technician findTechnician() { for (Technician t: technicians) { if (t.inState(Technician.AtServiceProvider)) { if (t.country == country){//serve customers living in the same country as the Service Provider return t;}}} return null; }
	sendToTechnician(ServiceRequest request, Technician t): void	Customized method developed to forward the customer requests to the available technician returned by calling the method findTechnician(). The method is defined as follows: void sendToTechnician(ServiceRequest request, Technician t) { t.request = request; send(request, t); }
	requestRecycler(TreatmentType type): void	Customized method developed to establish a communication with the Recycler agent to request the recycling of the washing machines. The method is defined as follows: void requestRecycler(TreatmentType type) { TreatmentRequest request = new TreatmentRequest (this, type); send(request, Recycler); } The Java keyword new creates a new instance of the agent type TreatmentRequest by indicating first the reference to the ServiceProvider agent that sends the request, and then the request type (i.e., Recycle). The Java keyword this is used to return the reference to the ServiceProvider agent that sends the request.
Agent links and communications	Bi-directional links with the Customer agent, Manufacturer agent, Recycler agent, and Product agent. These links allow agent communication via message passing and inter-agent method calls. In addition, to transfer the ServiceRequest agents created at model runtime by the Customer agent to the enter block of the DE process flowchart of the ServiceProvider agent, the built-in AnyLogic method take(agent) is called in the agent communication link as follows: if(msg instanceof ServiceRequest){ Incoming_CustomerRequests.take((ServiceRequest)msg); } Whereas, to transfer the Product agents created at model runtime by the Manufacturer agent to the enter block of the DE process flowchart of the ServiceProvider agent, the built-in AnyLogic method take(agent*)* is called in the agent communication link as follows: if(msg instanceof Product){ Incoming_Products.take((Product)msg); }	

### Technician agent

**Fig. 6:**
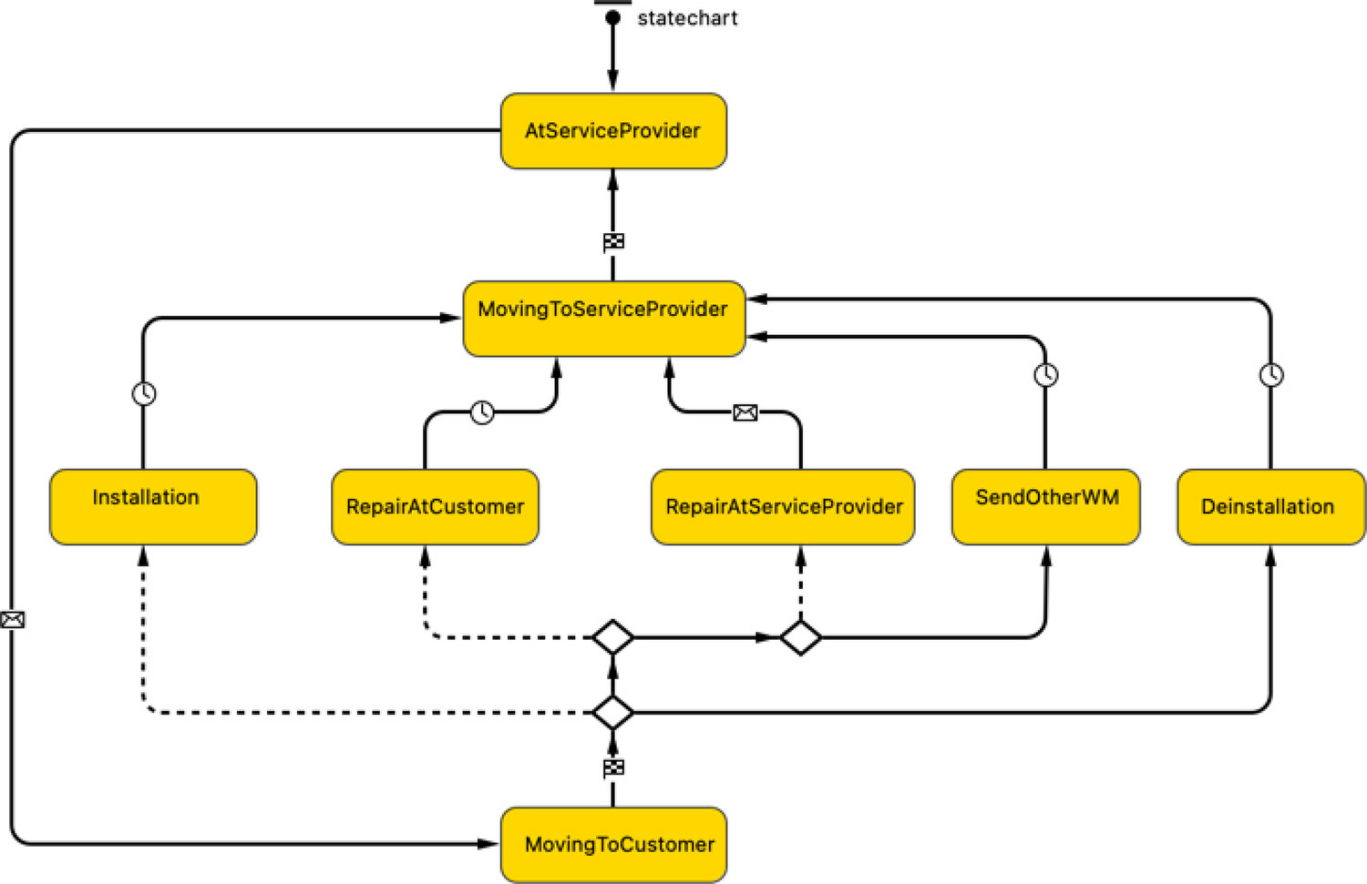
Agent-based statechart to simulate technician agent.

In the model, all technicians reside at the service provider's location, where they wait for requests to be processed. To define the behaviour of the Technician agent, the statechart shown in Fig. 6 is developed as a sequence of states. The technician initially waits for requests at the service provider's location. Once the request is received, the technician moves to the customer that placed the request. On reaching the customer, the technician begins the service deployment process, which, depending on the request type, could be installation, repair and maintenance, or deinstallation. After operation completion, the technician moves back to the service provider to fulfil the next request.

The main attributes, methods, and agent links used to develop the model logic of the Technician agent are reported in Table 3.

**Table 3:** Main attributes, methods, and agent links of the Technician agent.

Type	Name	Description
Attributes	country: String	Geographical location of the technician indicating the country
	region: String	Geographical location of the technician indicating the region
	sp: ServiceProvider	Service provider the technician is allocated to of type ServiceProvider
Methods	moveTo(Agent agent): void	Built-in AnyLogic method used to start the movement in the direction of the given target agent. This method is called to move the Technician agent to the Customer agent and back to the ServiceProvider agent. With this method, the agent will move along the routes of a GIS map's existing transportation network. It is assumed that the technician uses a light commercial vehicle (e.g., van) as means of transportation.
	deliverWashingMachine (ServiceRequest request, Product wm): void	Customized method developed to deliver the specified washing machine to the Customer agent that places a subscription request. The method is defined as follows: void deliverWashingMachine(ServiceRequest request, Product wm) { wm.request = request; wm.requestType = request.type; send(request,wm); }
	send(Object msg, Agent dest): void	Built-in AnyLogic function used to send a message to a given agent by indicating the parameters msg, i.e., the message, and dest, i.e., the destination agent.This method is called to send a message to the Customer agent after operation completion: • send("Installed", request.customer) when leaving the Installation state. • send("Repaired", request.customer) when leaving the RepairAtCustomer state • send("Deinstalled", request.customer) when leaving the Deinstallation state. In addition, this method is called when the Technician agent is in the RepairAtServiceProvider state to request the ServiceProvider agent to send another washing machine while this one is being repaired.if(request.customer.promptService == true)agentLink.send(request,sp);Note that an agent link is added to establish bi-directional communication between the Technician agent and the ServiceProvider agent.
	sendToWashingMachine (TreatmentRequest treatment, Product wm): void	Customized method developed to communicate with the statechart of the Product agent to define its journey. This method is called within the method requestTreatment(TreatmentTypetreatmentType,Techniciantechnician)*.* The method is defined as follows: void sendToWashingMachine(TreatmentRequest treatment, Product wm){ wm.treatment=treatment; wm.treatmentType = treatment.type; send(treatment, wm); }
	requestTreatment(TreatmentType treatmentType, Technician technician): void	Customized method developed to communicate with the statechart of the Product agent to define its journey.The method is defined as follows:void requestTreatment(TreatmentType treatmentType, Technician technician) {TreatmentRequest treatment = new TreatmentRequest (this, treatmentType);sendToWashingMachine(treatment, wm);wm.technician = technician;}This method is called on the following occasions: • When the Technician agent is the RepairAtServiceProvider state: requestTreatment(Repair, this); • When the Technician agent is in the DeinstallIation state: requestTreatment(Refurbish, this);
Variables	wm: Product	Variable used during model runtime to store the washing machine under service management.
	request: ServiceRequest	Variable used during model runtime to store the customer requests forwarded by the service provider.
Agent links and communications	Bi-directional links with the ServiceProvider agent, Customer agent, and Product agent. These links allow agent communications via message passing and inter-agent method calls.	

### Manufacturer agent

**Fig. 7:**
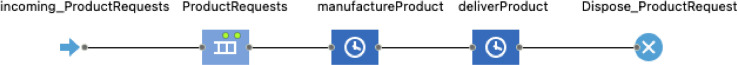
Discrete-event process flowchart to simulate the Manufacturer agent.

A discrete-event modelling methodology is employed to simulate the behaviour of the Manufacturer agent as this stakeholder deals mainly with manufacturing and logistics processes. The discrete-event process flowchart shown in Fig. 7 is developed to simulate the manufacturing and delivering process of new washing machines. When a product request is sent from the service provider, it enters the Incoming_ProductRequests block awaiting to be handled in the DE process flowchart.

The main attributes, methods, and agent links used to develop the model logic of the Manufacturer agent are reported in Table 4.

**Table 4:** Main attributes, methods, and agent links of the Manufacturer agent.

Type	Name	Description
Attributes	country: String	Geographical location of the manufacturer indicating the country
	region: String	Geographical location of the manufacturer indicating the region
	manufacturingCost: double	Manufacturing cost of the washing machine
	manufacturingCO2: double	Kg CO2-eq of due to manufacturing of the washing machine
	transportCost: double	Transport cost expressed in €/km/truck used to transport the washing machines to service providers’ location. It is assumed that a truck is used for the transportation of the washing machines. The truck transportation policy is full truckload.
	transportCO2: double	Transport kg CO2-eq expressed in kg CO2/tkm due to the transport of the washing machines to service providers’ location. It is assumed that a truck is used for the transportation of the washing machines. The truck transportation policy is full truckload.
Methods	add_washingMachines(): Product	Build-in AnyLogic method that creates a new agent of type Product and adds it into the population of washing machines. This method is called in the manufactureProduct block of the DE process flowchart.
	send(Object msg, Agent dest): void	Built-in AnyLogic method used to send a message to a given agent by indicating the message (msg) and the destination (dest) agent. This method is called to send a message to the Product agent as follows: send("Manufactured", wm).
Variables	wm: Product	Variable used during model runtime to store the reference to the washing machine manufactured.
Agent links and communications	Bi-directional links with the ServiceProvider agent and the Product agent. These links allow agent communications via message passing and inter-agent method calls. In addition, to transfer the ProductRequest agents created at model runtime by the ServiceProvider agent to the enter block of the DE process flowchart of the Manufacturer agent, the method take(agent) is called in the agent communication link as follows: if(msg instanceof ProductRequest){ incoming_ProductRequests.take((ProductRequest)msg); }	

### Recycler agent

**Fig. 8:**
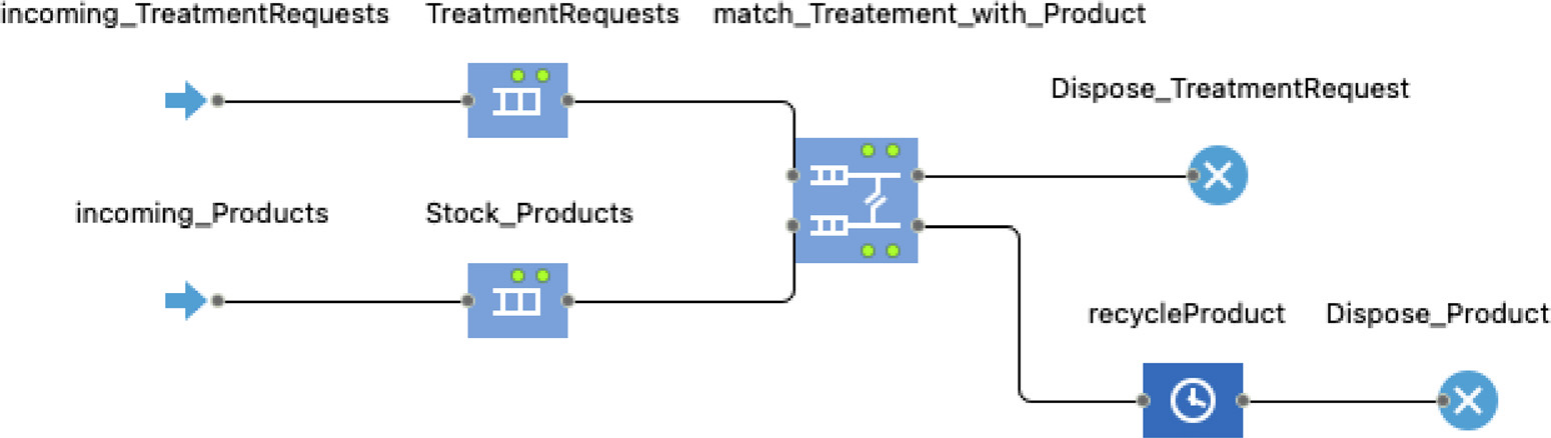
Discrete-event process flowchart to simulate the Recycler agent.

A discrete-event modelling methodology is employed to simulate the behaviour of the Recycler agent as this stakeholder deals mainly with reprocessing and logistics processes. The discrete-event process flowchart shown in Fig. 8 is developed to simulate the behaviour of the Recycler agent. When a treatment request and an end-of-life product are sent from the service provider, they enter the Incoming_TreatmentRequests and Incoming_Products blocks respectively awaiting to be handled in the DE process flowchart.

The main attributes, methods, and agent links used to develop the model logic of the Recycler agent are reported in Table 5.

**Table 5:** Main attributes, methods, and agent links of the Recycler agent.

Type	Name	Description
Attributes	country: String	Geographical location of the recycler indicating the country
	region: String	Geographical location of the recycler indicating the region
	recyclingCost: double	Recycling cost of the washing machine
	recyclingCO2: double	Kg CO2-eq due to recycling of the washing machine
Methods	remove_washingMachines(): void	Built-in AnyLogic method that removes a given agent of type Product from the population of washing machines.
	send(Object msg, Agent dest): void	Built-in AnyLogic method used to send a message to a given agent by indicating the message (msg) and the destination (dest) agent. This method is called to send a message to the Product agent as follows: send("Recycled", wm).
Variables	wm: Product	Variable used during model runtime to store the reference to the washing machine recycled.
Agent links and communications	Bi-directional links with the ServiceProvider agent and the Product agent. These links allow agent communications via message passing and inter-agent method calls. In addition, to transfer the TreatmentRequest agents created at model runtime by the ServiceProvider agent to the enter block of the DE process flowchart of the Recycler agent, the method take(agent)is called in the agent communication link as follows: if(msg instanceof TreatmentRequest){ incoming_TreatmentRequests.take((TreatmentRequest)msg); }	

### Product agent

**Fig. 9:**
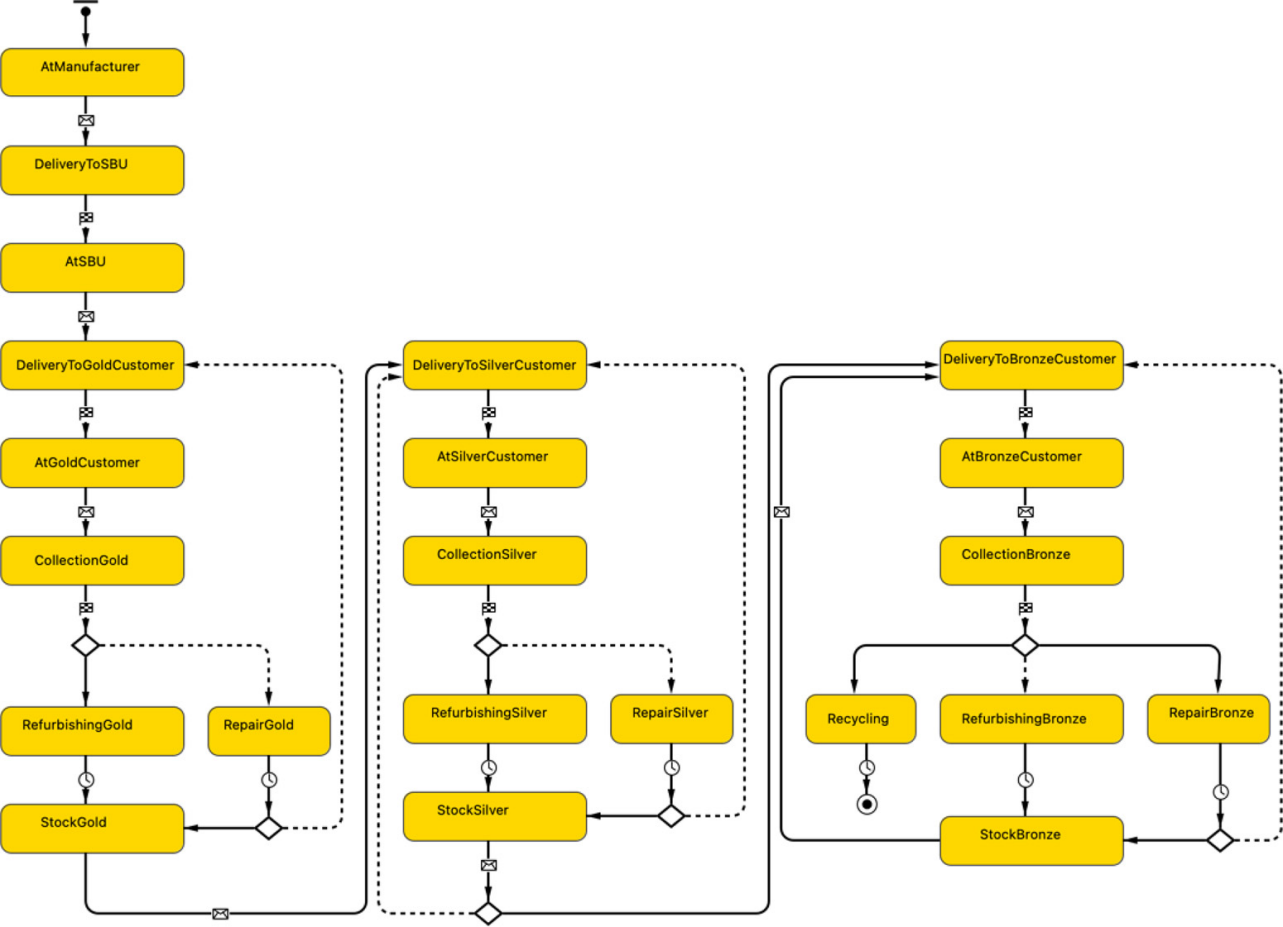
Agent-based statechart to simulate the product journey throughout its multiple lifecycles.

When the Manufacturer agent calls the method add_washingMachines() in the manufactureProduct block of the DE process flowchart shown in Fig. 7, a new agent of type Product is created and added to the population of washing machines. The agent-based statechart shown in Fig. 9 is developed to define the journey of the washing machine throughout its multiple lifecycles as a result of the product's interaction with the various CMS stakeholders from manufacturing, to distribution, product use phase, value recovery and re-distribution, to end-of-life treatment.

The main attributes, methods, and agent links used to develop the model logic of the Product agent are reported in Table 6.

**Table 6:** Main attributes, methods, and agent links of the Product agent.

Type	Name	Description
Attributes	ID: int	Unique product identifier number of type integer
	weight: double	Weight of the washing machine expressed in kg
	failureRate: double	Product failure rate of type double
	unit_Emissins: double	Average kg CO2-eq of a washing cycle
Methods	moveTo(Agent agent): void	Built-in AnyLogic method used to start the movement in the direction of the given target agent. This method is called to move the washing machine from one stakeholder to another in the circular manufacturing system by indicating the destination agent (e.g., ServiceProvider, Customer, Recycler).
	distanceByRoute(Agent agent): double	Built-in AnyLogic method used to calculate the distance from the agent under consideration to another one by route (measured in meters in case of GIS environment). This method is called to calculate the transport distance of the washing machine from one stakeholder to another in the circular manufacturing system
	send(Object msg, Agent dest): void	Built-in AnyLogic method used to send a message to a given agent by indicating the message (msg) and the destination (dest) agent. This method is called to establish a connection between the agent-based statechart of the Product agent and the discrete-event process flowchart of the ServiceProvider agent as follows: agentLink.send(this, ServiceProvider); Note that agentLink is used to create a bi-directional link between the Product agent and the ServiceProvider agent. The Java keyword this is used to return the reference to the Product agent. In this way, the washing machine enters the Incoming_Products enter block of the DE process flowchart within the ServiceProvider agent. Thus, this method is called when the Product agent exits the states RefurbishingGold, RefurbishingSilver, and RefurbishingBronze.
	time(): double	Built-in AnyLogic method that returns the current model time in model time units (i.e., months). This method is called to account for the time a washing machine spends at each customer location. Thus, it is called when the washing machine is in the states AtGoldCustomer, AtSilverCustomer, and AtBronzeCustomer.
	defineRefurbishCost(Product wm): double	Customized method built to define the refurbish cost. This cost can be calculated by taking into account the service life of the washing machine and/or the total number of washing cycles performed by the washing machine. For instance, the refurbished cost after the first use cycle could be defined as follows: double defineRefurbishCostGold(Product wm) { double refurbishingCost; if (wm.serviceLife>60){ refurbishingCost = RefurbishCost + CleaningCost + RepackagingCost; refurbishGold=true;} else refurbishingCost = CleaningCost + RepackagingCost; return refurbishingCost; }
Variables	manufacturingCost: double	Variable used during model runtime to store the manufacturing cost of a washing machine.
	forwardTransportDistance: double	Variable used during model runtime to store the transport distance of a washing machine from the Manufacturer agent to the ServiceProvider agent.
	forwardTransportCost: double	Variable used during model runtime to store the transport cost of a washing machine from the Manufacturer agent to the ServiceProvider agent based on the distance travelled and the unit transport cost.
	forwardTransportCO2: double	Variable used during model runtime to store the transport CO2 of a washing machine from the Manufacturer agent to the ServiceProvider agent based on the distance travelled and the unit transport CO2.
	forwardTransportServiceDistance: double	Variable used during model runtime to store the transport distance of a washing machine from the ServiceProvider agent to the Customer agent based on the distance travelled.
	forwardTransportServiceCost: double	Variable used during model runtime to store the transport cost of a washing machine from the ServiceProvider agent to the Customer agent based on the distance travelled and the unit transport cost.
	forwardTransportServiceCO2: double	Variable used during model runtime to store the transport CO2 of a washing machine from the ServiceProvider agent to the Customer agent based on the distance travelled and unit transport CO2.
	installationCost: double	Variable used during model runtime to store the installation cost of a washing machine based on transport cost and technician labour cost.
	repairCost: double	Variable used during model runtime to store the repair cost of a washing machine based on transport cost, labour cost, and spare parts replacement.
	reverseTransportServiceDistance: double	Variable used during model runtime to store the transport distance of a washing machine from the Customer agent to the ServiceProvider agent based on the distance travelled.
	reverseTransportServiceCost: double	Variable used during model runtime to store the transport cost of a washing machine from the Customer agent to the ServiceProvider agent based on the distance travelled and the unit transport cost.
	reverseTransportServiceCO2: double	Variable used during model runtime to store the transport CO2 of a washing machine from the Customer agent to the ServiceProvider agent based on the distance travelled and the unit transport CO2.
	deinstallationCost: double	Variable used during model runtime to store the deinstallation cost of a washing machine based on labour cost and transport cost.
	refurbishCost: double	Variable used during model runtime to store the refurbish cost of a washing machine.
	reverseTransportDistance: double	Variable used during model runtime to store the transport distance of a washing machine from the ServiceProvider agent to the Recycler agent based on the distance travelled.
	reverseTransportCost: double	Variable used during model runtime to store the transport cost of a washing machine from the ServiceProvider agent to the Recycler agent based on the distance travelled and the unit transport cost.
	reverseTransportCO2: double	Variable used during model runtime to store the transport CO2 of a washing machine from the ServiceProvider agent to the Recycler agent based on the distance travelled and the unit transport CO2.
	recyclingCost: double	Variable used during model runtime to store the refurbish cost of a washing machine.
	durationUseCycle: double	Variable used during model runtime to store the duration of each usecycle a washing machine goes through during its entire lifetime.
	nrUseCycles: double	Variable used during model runtime to store the number of usecycle a washing machine goes through during its entire lifetime.
	nrWashingCycles: double	Variable used during model runtime to store the number of washing cycles a washing machine goes through during its entire lifetime.
	serviceLife: double	Variable used during model runtime to store the accumulated period expressed in months the washing machine has been in use/service
	usePhaseCO2: double	Variable used during model runtime to store the kg CO2-eq during the use phase of the washing machine as a result of the number of washing cycles performed by the washing machine.
	LCC: double	Variable used during model runtime to store the total lifecycle cost of a washing machine.
	LCI: double	Variable used during model runtime to store the total lifecycle environmental impact of a washing machine.
	LCR: double	Variable used during model runtime to store the total lifecycle revenues of a washing machine.
Agent links & communications	Bi-directional links with the Manufacturer agent, ServiceProvider agent, Customer agent, and Recycler agent. These links allow agent communication via message passing and inter-agent method calls.	

**Fig. 10:**
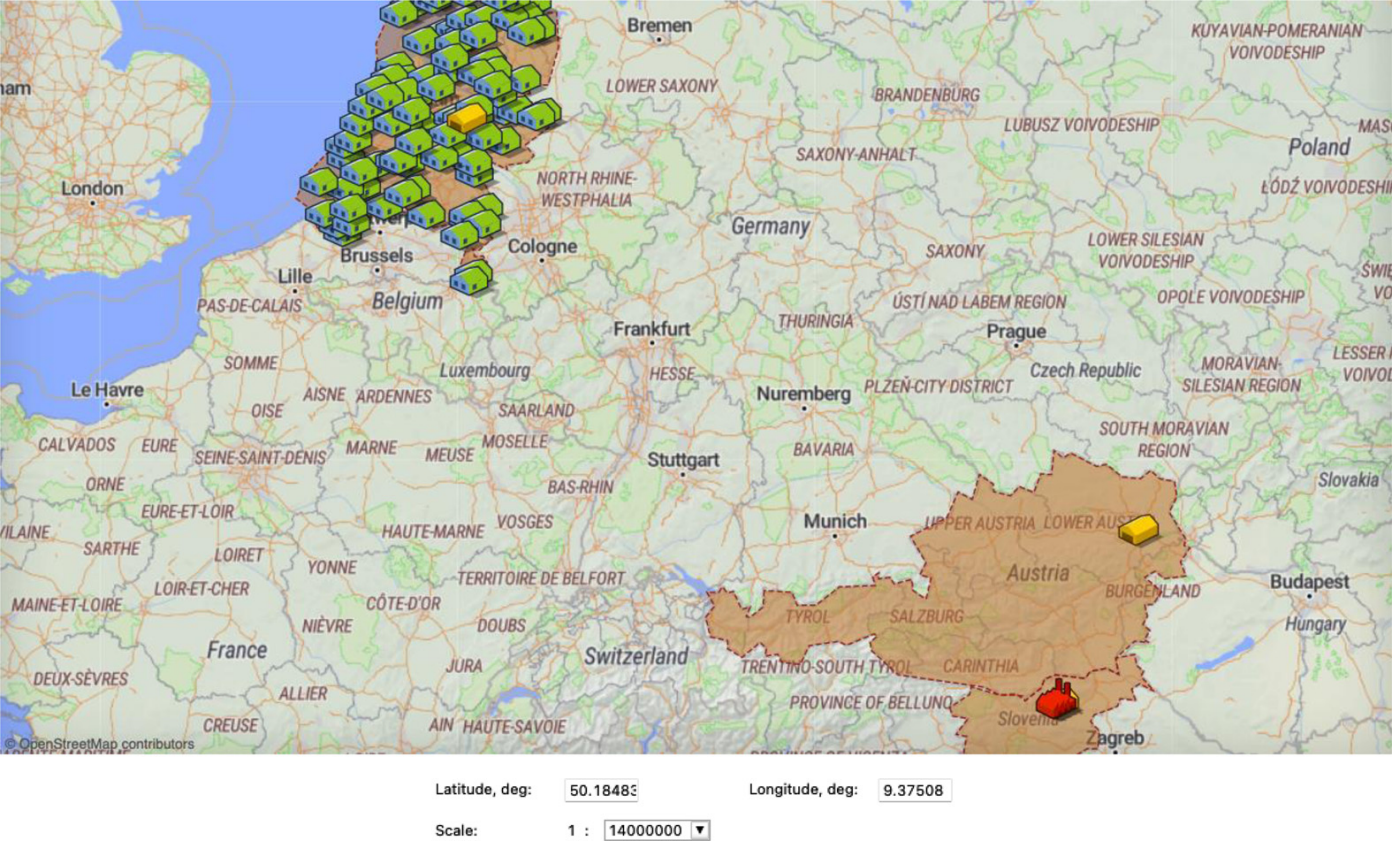
Stakeholder-agents placed into a geospatial environment defined with a Geographic information System (GIS) map.

After defining the behaviour of the CMS stakeholders and their interrelations, the simulation environment in which the stakeholder-agents will be placed is selected. The different stakeholder-agents are placed into a geospatial environment defined with a GIS map as shown in Fig. 10 depending on the stakeholders’ geographical location. Thus, by considering real-life locations of the stakeholders, it is possible to retrieve real distances travelled between CMS stakeholders to better estimate transport cost and greenhouse gas emissions.

Once the model logic is built, one can run the simulation model to obtain statistics both at the agent level and at the system level using the values of the variables defined in Table 1, Table 2, Table 3, Table 4, Table 5, Table 6. As the CMS multi-method simulation model allows to adopt a lifecycle perspective, the lifecycle costs, lifecycle revenues, and lifecycle environmental impact of deploying multiple-lifecycles washing machines in a subscription-based scheme are estimated. In addition, the quality, quantity and timing of product return flows are monitored over time. Thus, the CMS multi-method simulation model can be used as a decision support tool to design CMS that are feasible in both economic and environmental terms. In Roci et al. [8] the results of the multi-method simulation model developed for the white goods manufacturer are presented.

## Method verification and validation

According to Sterman [11] “no model can be verified or validated because all models are wrong”. This approach implies that all models are limited, simplified representations of the real world. However, some level of verification and validation is necessary to ensure that the model is an accurate representation of the real-world system and it generates reasonable results. The approaches used for verification and validation of simulation models, in general, can be used to verify and validate the CMS multi-method simulation model. In this paper, the CMS multi-method simulation model has been analysed and validated using the validation techniques presented in Sargent [9] Thus, animation, comparison to other models (e.g., analytical models), degenerate tests and extreme condition tests, and face validity were employed to verify and validate the model. To ensure correct implementation of computer code, tests have been performed during the implementation of the sub-models (e.g., using specific parameter settings expected behavioural outcomes have been verified). Moreover, the AnyLogic platform used to implement the CMS multi-method simulation model allows embedding model animations, hence, checking for correct implementation of the model logic. In addition, the model for the case study has been developed in close collaboration with the white goods manufacturer and the model results have been discussed closely with representatives from the white goods manufacturer and their service providers.

## Declaration of Competing Interest

The authors declare that they have no known competing financial interests or personal relationships that could have appeared to influence the work reported in this paper.
